# Oxidative Stress (Glutathionylation) and Na,K-ATPase Activity in Rat Skeletal Muscle

**DOI:** 10.1371/journal.pone.0110514

**Published:** 2014-10-13

**Authors:** Carsten Juel

**Affiliations:** Department of Biology, University of Copenhagen, Copenhagen, Denmark; University of California, Davis, United States of America

## Abstract

**Background:**

Changes in ion distribution across skeletal muscle membranes during muscle activity affect excitability and may impair force development. These changes are counteracted by the Na,K-ATPase. Regulation of the Na,K-ATPase is therefore important for skeletal muscle function. The present study investigated the presence of oxidative stress (glutathionylation) on the Na,K-ATPase in rat skeletal muscle membranes.

**Results:**

Immunoprecipitation with an anti-glutathione antibody and subsequent immunodetection of Na,K-ATPase protein subunits demonstrated 9.0±1.3% and 4.1±1.0% glutathionylation of the α isoforms in oxidative and glycolytic skeletal muscle, respectively. In oxidative muscle, 20.0±6.1% of the β1 units were glutathionylated, whereas 14.8±2.8% of the β2-subunits appear to be glutathionylated in glycolytic muscle. Treatment with the reducing agent dithiothreitol (DTT, 1 mM) increased the in vitro maximal Na,K-ATPase activity by 19% (P<0.05) in membranes from glycolytic muscle. Oxidized glutathione (GSSG, 0–10 mM) increased the in vitro glutathionylation level detected with antibodies, and decreased the in vitro maximal Na,K-ATPase activity in a dose-dependent manner, and with a larger effect in oxidative compared to glycolytic skeletal muscle.

**Conclusion:**

This study demonstrates the existence of basal glutathionylation of both the α and the β units of rat skeletal muscle Na,K-ATPase. In addition, the study suggests a negative correlation between glutathionylation levels and maximal Na,K-ATPase activity.

**Perspective:**

Glutathionylation likely contributes to the complex regulation of Na,K-ATPase function in skeletal muscle. Especially, glutathionylation induced by oxidative stress may have a role in Na,K-ATPase regulation during prolonged muscle activity.

## Introduction

Ion gradients across the skeletal muscle membrane undergo pronounced perturbations during intense muscle contractions. These activity-induced changes in ion distribution affect muscle excitability and may lead to impairment of force development (muscle fatigue). The Na,K-ATPase ( = the Na,K-pump) counteracts the rundown of transmembrane gradients for Na^+^ and K^+^ during muscle activity. Regulation of Na,K-ATPase is therefore of importance for muscle function. It is generally accepted that the Na,K-ATPase is regulated during muscle activity by a multifactorial process that includes phosphorylation, sensitivity to hormones, and changes in the intracellular Na^+^ concentration [1]. Furthermore, purinergic stimulation of the Na,K-ATPase may be involved [2].

Reactive oxygen species are generated in skeletal muscle during activity [3], [4]. Oxidative stress may lead to chemical modification of muscle proteins of importance for muscle function. The oxidative modifications involve the formation of reversible disulphide bonds between glutathione and reactive cysteine thiols (S-glutathionylation). Glutathionylation (oxidative stress) has been demonstrated to increase contractile apparatus Ca^2+^ sensitivity in rats and humans [5], and it has been reported that glutathionylation of Na,K-ATPase proteins may lead to modifications in Na,K-ATPase function in heart muscle [6], [7], [8]. Glutathionylation has been reported to involve the β1 isoform [7], [9], the α-subunits [10], [11], and the regulatory protein phospholemman (PLM, FXYD1) [12]. Similar glutathionylation may be present in skeletal muscle. It is therefore hypothesized that glutathionylation affects Na,K-ATPase function in skeletal muscle.

The first aim of the present study was to quantify the basal glutathionylation level of Na,K-ATPase isoforms in rat muscle obtained at rest. The second aim was to investigate if in vitro glutathionylation affects Na,K-ATPase activity in purified rat muscle membranes.

## Materials and Methods

### Ethical Approval

The animal handling was conducted in accordance with the Danish Animal Welfare Regulations. The animal study was approved (P13-073) by the Department of Experimental Medicine – International Animal Care and Use System. Male Wistar rats (body weight 120–150 g) were provided with ad libitum food and water and kept under a 12/12-h dark/light cycle. The rats were killed with a blow to the neck followed by cervical dislocation, and muscle tissue was immediately removed and frozen.

### Treatment of samples

Rat muscle (white vastus lateralis or red gastrocnemius) were homogenized for 30 s (Polytron PT 2100) in 250 mM mannitol, 30 mM L-histidine, 5 mM EGTA and 0.1% deoxycholate, adjusted to pH 6.8 with Tris-base. This homogenate was used for immunoprecipitation and Western blotting. Part of the homogenate was centrifuged at 3000×*g* for 30 min, and the resulting supernatant was centrifuged at 190,000×*g* for 90 min (at 4°C). The final pellets (called the 190,000×*g* fraction) were used for the Na,K-ATPase assay. The protein content of samples was determined in triplicate using a bovine serum albumin standard (DC protein assay; Bio-Rad, Richmond, CA).

### Na,K-ATPase assay

Na^+^-stimulated Na,K-ATPase activity was determined by measuring ATP hydrolysis. Released inorganic phosphate (P_i_) was detected using a malachite-based Biomol Green reagent (Biomol AK-111, Enzo Life Sciences) as previously described [13]. Samples (5 µg protein) were suspended in 70 µL assay buffer (10 mM KCl, 5 mM MgCl_2_, 50 mM Tris-base, 5 mM EGTA, pH 7.4). Na^+^ was added to the samples to a final concentration of 0, 2, 4, 6, 10, 20, 40 and 80 mM (the ionic strength was kept constant by substituting NaCl with choline chloride). After 5 min of pre-incubation at 37°C, the reaction was started by adding Mg-ATP to a final concentration of 0.5 mM. After 30 min, the reaction was terminated by adding 1 mL Biomol Green reagent at room temperature. After 30 min incubation, absorbance was read at 620 nm and [P_i_] was calculated from a standard curve. All samples were run in duplicate (0 mM Na^+^ was measured four times), and the ATPase activity at 0 mM Na^+^ was subtracted from all of the activity values. Previous experiments demonstrated that Na^+^-stimulated activity was completely inhibited by pre-incubation with 2 mM ouabain [14]. The Na,K-ATPase assay could only be applied to the 190,000×*g* fraction, due to the inevitable high background Ca^++^-ATPase activity in unpurified samples. In some experiments, changes in Na,K-ATPase activity were evaluated by the difference in activity at 0 and 40 mM Na^+^.

### Quantification of glutathionylation

Western blotting of homogenized muscle material has shown that many proteins are susceptible to glutathionylation, including other proteins with the same molecular weight as the Na,K-ATPase α-subunit [5]. To study the glutathionylation of Na,K-ATPase subunits, it is therefore necessary to use a purification step to isolate the subunits. Glutathionylated proteins were immunoprecipitated with the anti-GSH antibody. The precipitate was subjected to Western blotting, and the Na,K-ATPase isoforms detected with α- and β-subunit antibodies. The level of glutathionylated α- and β-subunit was evaluated by calculating the ratio between isoform detection after immunoprecipitation (with anti-GSH) and the total amount of that subunit detected in the homogenate with the same isoform-specific antibody.

### Immunoprecipitation

Muscle homogenate (200 µg of protein) was incubated in ice-cold lysis buffer (100 mM NaCl, 20 mM Tris-base, 10 mM NaF, 1 mM PMSF, 1 mg/ml C_12_E_8_, pH 7.4) and the anti-GSH antibody (5 µg antibody/200 µg total protein), incubated overnight at 5°C with end-over-end rotation. Spinning at 20,000×*g* for 20 min was applied to remove the non-lysed fraction. The supernatant was mixed with 15 µl packed protein G agarose beads (16-266, Millipore). After 4 h of incubation, the beads were sedimented, and then washed five times with lysis buffer. Sample buffer was added and the mixture was heated to 56°C for 20 min, sedimented and the supernatant used for immunoblotting.

### Western blotting

Samples were mixed with sample buffer (2 mM Tris-HCl, 0.2 mM EDTA, 20 mM DTT, 4% SDS, 10% glycerol, 0.04% bromophenol blue, pH 8.0). Equal amounts of protein were loaded into each lane and separated by 8–18% SDS–PAGE (Excel 8–18% gradient gel, Amersham). The proteins were then electroblotted onto a polyvinylidene difluoride membrane (Immobilon-P, Millipore). The membrane was blocked for 60 min at room temperature in TS-buffer (10 mM Tris-Base, 0.9% NaCl, pH 7.4) containing 1% BSA, 2% skimmed-milk powder, and 0.1% Tween-20 before incubation with primary antibody diluted in a similar buffer overnight (4°C). After treatment with a horseradish-peroxidase-coupled secondary antibody (Dako, Denmark) for 60 min at room temperature, the membrane was repeatedly washed in TS-buffer with 0.05% Tween-20 and finally in TS-buffer without Tween-20. The membrane was incubated with enhanced chemiluminescence reagent (ECL-prime, Amersham) and visualized in an ImageQuant LAS 4000. Samples to be compared were loaded on the same gel. The Image station also produced a gel picture to visualize the molecular weight marker. Relative protein concentrations were quantified by the image station software using background subtraction.

### Antibodies

Immunoprecipitation of glutathionylated proteins was carried out with a mouse monoclonal anti-GSH antibody at a concentration of 5 µg antibody/200 µg total protein (101-A, clone D8, Virogen, USA). All α-subunits were immunodetected with the rabbit monoclonal sc-28800 diluted 1:1000 (H-300, Santa Cruz Biotech). The β1 isoform was immunodetected with a polyclonal rabbit antibody generously provided by Dr Pedersen, University of Copenhagen. The β2 isoform was detected with the rabbit polyclonal antibody 06-1711 diluted 1:2000 (Millipore, USA).

### Chemicals

Oxidized glutathione, GSSG, was from Sigma-Aldrich (G4626).

### Statistics

V_max_ for Na^+^ stimulated ATPase activity was determined for each experiment by non- linear regression (Sigma Plot software) with a Hill equation. Mean V_max_ values for each group of experiments were compared with Student’s paired t-test (Sigma Plot software). For the gel data: Mean values are compared using Student’s paired t-test (control vs. treatment). P<0.05 was considered significant.

## Results

### Glutathionylation level in rat muscle samples

**Figure 1:**
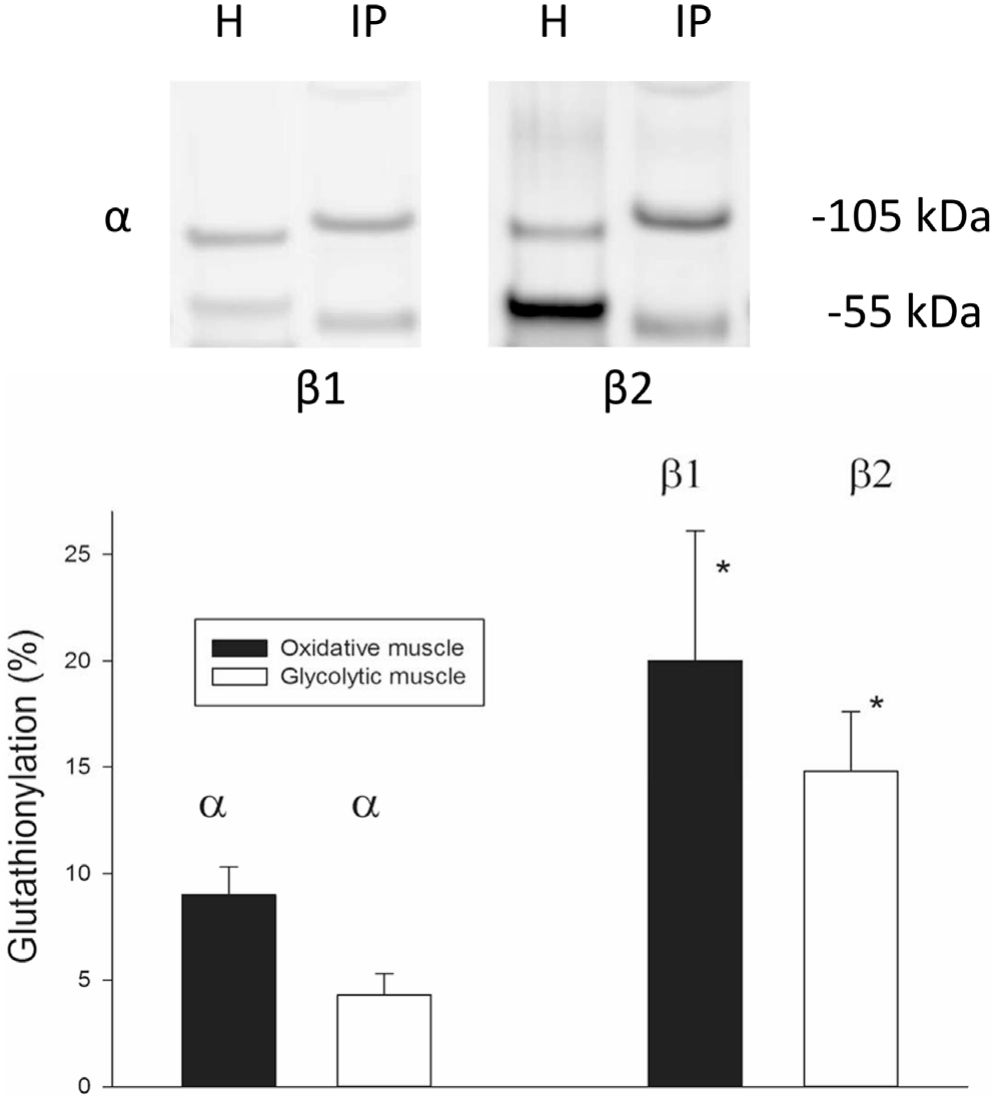
Glutathionylation in untreated muscle. Upper panel: Examples of Western blots of Na,K-ATPase α, β1 and β2 isoforms in homogenates and in the immunoprecipitate obtained with anti-GSH antibodies. The fraction of glutathionylated proteins was only 5–20%. To get a clear signal it was therefore necessary to immunoprecipitate from a larger (20 fold) amount of protein than used for the homogenate sample (which was probed for the total subunit content). H: isoforms detected in untreated muscle homogenate, 10 µg protein per lane, IP: isoforms detected in immunoprecipitate from 200 µg protein. Two antibodies were used successively on the same gel; first anti-β then anti-α. The figure bands originate from two gels. Pictures of uncropped gels and identification criteria are provided in file S1. Lower panel: Homogenates from oxidative and glycolytic muscle were immunoprecipitated with an anti-GSH antibody and the precipitate immunoblotted with Na,K-ATPase isoform antibodies. The glutathionylation level was calculated relative to immunoblotted crude muscle homogenates. *: significantly different from the fraction of α isoform in the same muscle type. Mean + SEM shown, one oxidative and one glycolytic muscle from each of 6 animals. All samples run in duplicate.

The basal levels of glutathionylation of Na,K-ATPase α- and β-subunits were quantified in the precipitate obtained in immunoprecipitation experiments with an anti-GSH antibody. The levels of glutathionylated α-subunits were measured in both oxidative and glycolytic muscle. Since the distributions of β isoforms are strongly fibre-type dependent in rat muscle [14], different muscles were used to quantify basal glutathionylation of β1 and β2 subunits. The oxidative red gastrocnemius muscle was used for quantification of the glutathionylated β1 isoform and the glycolytic vastus lateralis muscle was used for quantification of the glutathionylated β2 isoform (Figure 1, upper panel).

For the oxidative muscle, the fraction of immunoprecipitated glutathionylated α-subunits was 9.0±1.3% of the total amount of α-isoform protein in muscle homogenates. For the glycolytic muscle, the fraction was 4.3±1.0%. In the oxidative muscle, 20.0±6.1% of the β1 isoform was immunoprecipitated with the anti-GSH antibody, whereas in the glycolytic muscle, 14.8±2.8% of the β2 isoform was immunoprecipitated (Figure 1, lower panel).

The possibility exists that some of the glutathionylation takes place during the preparation procedure. Samples were therefore incubated with 0.2 mM iodoacetic acid (which reacts with free thiol-groups) during homogenization and when the samples were not frozen. In experiments similar to Figure 1, the glutathionylation level was 116±12%, 134±22, and 110±18% of control for α, β1, and β2 subunits, respectively (mean ± SE, 10 samples, 2 from each rat). For both α-, β1, and β2 subunits, the glutathionylation levels in iodoacetic-acid-treated samples were not significantly different from control.

### Effect of dithiothreitol

**Figure 2:**
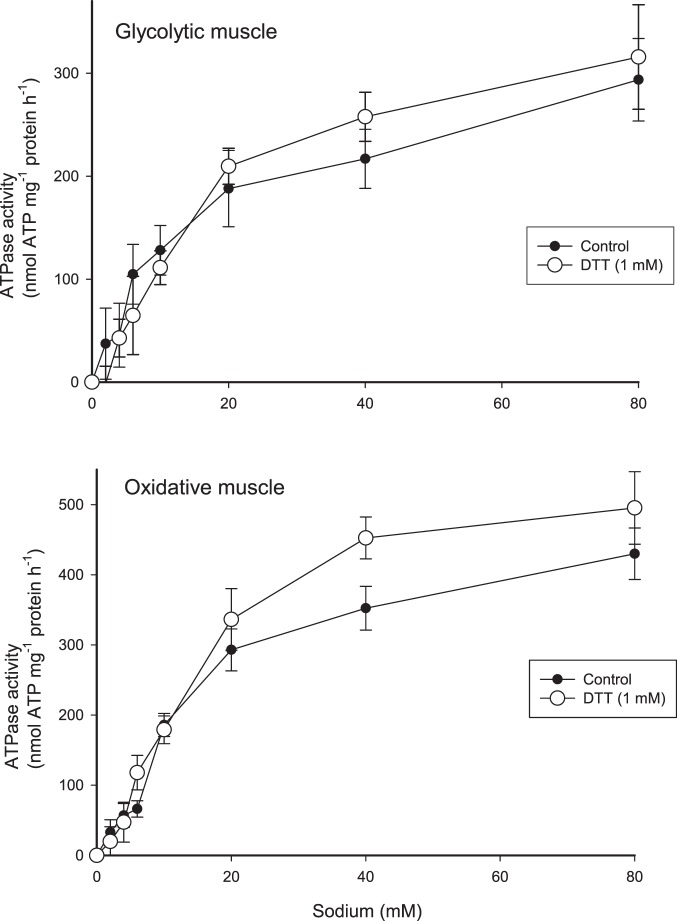
Effect of 1 mM DTT (dithiothreitol) on Na^+^-dependent Na,K-ATPase activity in purified muscle membranes from oxidative (lower panel) and glycolytic (upper panel) rat muscle. One sample from each rat was measured at each Na^+^ concentration, the values therefore represent the mean from eight rats (Mean ± SEM shown, n = 8). Closed symbols: control. Open symbols: 1 mM DTT. For the oxidative muscle the mean V_max_ with DTT was significantly higher than control (P<0.05).

Dithiothreitol (DTT) is a reducing agent, which is expected to decrease existing glutathionylation. The effect of 1 mM DTT on maximal Na^+^-dependent Na,K-ATPase activity was measured in purified membranes (the 190,000×*g* fraction) from oxidative and glycolytic rat muscle. DTT increased mean V_max_ for Na,K-ATPase activity in membranes from oxidative muscle from 456±35 to 543±42 nmol ATP mg^−1^ protein h^−1^(n = 8, P<0.05). Similar experiments with membranes from glycolytic muscle showed no effect of DTT (405±43 vs. 429±58 nmol ATP mg^−1^ protein h^−1^ (Figure 2).

### GSSG-induced in vitro glutathionylation

**Figure 3:**
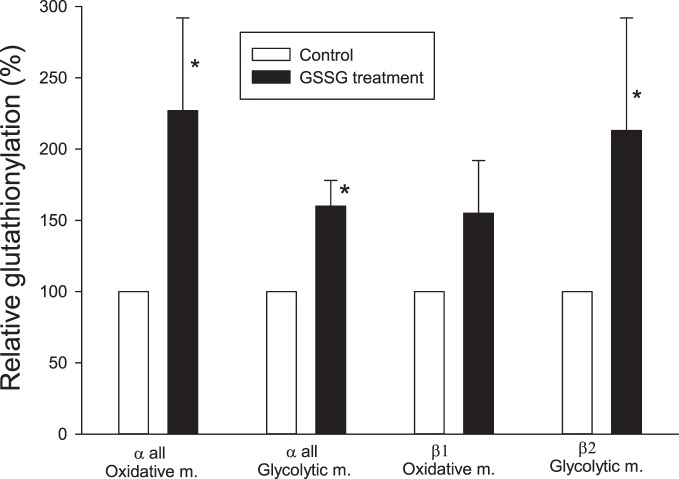
GSSG-induced changes in relative glutathionylation level. Rat muscle homogenates from oxidative and glycolytic muscle were incubated in 5 mM GSSG for 20 min, immunoprecipitated with an anti-GSH antibody, and the Na,K-ATPase subunits were quantified by Western blotting. Examples of gel pictures are provided in the S1 file. The glutathionylation was calculated relative to the control. n = 6 in all groups. *: significantly different from control (P<0.05). The difference for β1 oxidative muscle is not different (P = 0.05).

To investigate a possible causative correlation between glutathionylation level and Na,K-ATPase function, the effect of GSSG treatment on glutathionylation was investigated. Rat muscle homogenates from oxidative and glycolytic muscle were incubated in 5 mM GSSG for 20 min, immunoprecipitated with an anti-GSH antibody and the Na,K-ATPase subunits quantified by Western blotting. The changes in glutathionylation were calculated relative to control (Figure 3). GSSG treatment significantly increased glutathionylation of α(all) both in oxidative and glycolytic muscle. In addition GSSG significantly increased glutathionylation of β2 proteins in glycolytic muscle, whereas no significant effect was obtained for β1 proteins in oxidative muscle.

### GSSG-induced changes in Na,K-ATPase activity

**Figure 4:**
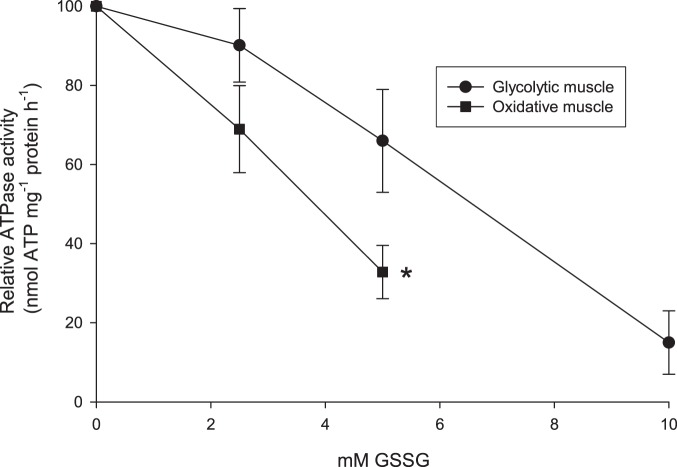
Effect of pre-treatment with oxidized glutathione (GSSG) on Na,K-ATPase activity in purified membranes from oxidative and glycolytic muscle. The Na,K-ATPase activity was evaluated by the difference in ATPase activity at 0 and 40 mM Na^+^. Values are calculated relative to control. Muscles from 6 rats. Squares: oxidative muscle, round symbol: glycolytic muscle *: Na,K-ATPase activity with 5 mM GSSG was significantly lower in oxidative muscle compared to glycolytic muscle (P<0.05). Na,K-ATPase activity with 2.5 mM GSSG tended to be lower in oxidative compared to glycolytic muscle (P<0.1).

Rat muscle samples (190,000×*g* fraction) were incubated in oxidized glutathione (GSSG, 0–10 mM) for 20 min at 37°C, and the Na,K-ATPase activity was measured at 37°C and compared to the activity in untreated control samples. The changes in Na,K-ATPase activity were evaluated by the difference in activity between 0 and 40 mM Na^+^. GSSG decreased the Na,K-ATPase activity in a dose-dependent manner (Figure 4). The inhibitory effect of 5 mM GSSG was significantly higher in oxidative muscle compared to glycolytic muscle (P<0.05).

## Discussion

This study demonstrates the existence of a basal glutathionylation of rat muscle Na,K-ATPase subunits. In addition, glutathionylation was demonstrated to reduce Na,K-ATPase activity. The first finding is based on immunoprecipitation experiments with an anti-GSH antibody and on the finding that pre-treatment with the reducing agent DTT increased the Na,K-ATPase activity. The second finding is based on the in vitro glutathionylation, which can be induced with oxidized glutathione (GSSG), and on the finding that GSSG reduced the maximal Na,K-ATPase activity in a dose-dependent manner. Furthermore, the study revealed that oxidative muscle is more sensitive to glutathionylation regulation than glycolytic muscle.

### Basal glutathionylation

Immunoprecipitation with an anti-GSH antibody suggests a basal glutathionylation of Na,K-ATPase subunits. The glutathionylation level of the α-subunits was low (4–9%), whereas the β1- and β2–subunits were 20% and 15% glutathionylated in oxidative and glycolytic muscle, respectively (Figure 1). However, these values should be taken with caution, as co-immunoprecipitation may take place [7], indicating that an overestimation of the glutathionylation could be the result. On the other hand, although the GSH antibody concentration used for the immunoprecipitation studies was higher than that recommended by the suppliers, a complete immunoprecipitation is unlikely to have occurred. In spite of these reservations, it seems justified to conclude that the glutathionylation of the β-subunits is higher than that of the α-subunits.

The experiments with iodoacetic acid pre-treatment exclude the possibility that glutathionylation takes place during the sample preparation procedure.

### Effect of dithiothreitol

To allow quantification of the Na,K-ATPase activity experiments were carried out with purified membranes to reduce the background Ca^++^-ATPase activity. Dithiothreitol (DTT, 1 mM) increased the maximal in vitro Na,K-ATPase activity in purified muscle membranes from oxidative muscle (Figure 2). Since DTT treatment has been shown to reduce basal glutathionylation [7], [10], the present finding supports the existence of a basal reversible glutathionylation and that maximal Na,K-ATPase activity is reduced by glutathionylation in the control samples.

### Changes in Na,K-ATPase activity

We tested the effect of 5 mM oxidized glutathione (GSSG) on subunit glutathionylation level, because this concentration had a marked influence on Na,K-ATPase activity (below). Pre-treatment of samples with 5 mM GSSG increased the glutathionylation level of both the α- and β2-isoforms (Figure 3). However, it must be repeated that co-immunoprecipitation is likely to take place with the technique used. Therefore, the exact sensitivity of the isoforms cannot be evaluated. Pre-treatment of samples with GSSG significantly reduced the maximal Na,K-ATPase activity in a dose-dependent manner (Figure 4). Taken together, these experiments suggest a causative reverse correlation between glutathionylation and Na,K-ATPase activity. High doses of GSSG (10 mM) almost removed Na,K-ATPase activity. The inhibitory effect of GSSG was significantly higher (nearly doubled) in membranes from oxidative compared to glycolytic fibres. It must be noted that the level of GSSG used in the present study is high compared to the concentration (1 mM) used in studies with myocardial cells [10], [11], although high concentrations (up to 10 mM) were used in a study with skeletal muscle [5].

There appears to be some controversy in the literature regarding α-subunit glutathionylation. Some studies report redox sensitivity of the Na,K-ATPase α-subunit in myocardial tissue [10], [11], whereas other studies found no evidence for glutathionylated α1-subunits [7], [9]. As noted above, the glutathionylation found in the α-subunits (Figure 1) may partly be due to co-immunoprecipitation with β-subunits [7]. Therefore, the α subunit glutathionylation in Figure 1 may be overestimated. Figure 2 demonstrates an effect of DTT on Na,K-ATPase activity only in oxidative muscle. In addition, the GSSG sensitivity was higher in oxidative compared to glycolytic muscle. These observations fit in with the presence of a high fraction of the β1 isoform in oxidative muscle [14], and are probably related to the presence of a reactive cysteine (Cys46) in the β1 isoform [7].

The present experiments demonstrated a basal Na,K-ATPase subunit glutathionylation of 5–20% in rat skeletal muscle samples. The theoretical maximal Na,K-ATPase activity can be calculated from the density of Na,K-ATPase proteins (for instance, determined with radiolabelled ouabain binding) and the turnover number. However, the finding of a basal glutathionylation indicates that some Na,K-ATPase proteins are not functional or has a reduced functional capacity. Therefore, if glutathionylation is not taken into consideration, such calculations could overestimate the maximal Na,K-ATPase activity.

The basal glutathionylation described in the present study suggests that reversible glutathionylation may be of importance in periods with increased oxidative stress. Future studies will show if glutathionylation-dependent inhibition of muscle Na,K-ATPase subunits has a regulatory role during muscle activity.

## Supporting Information

File S1 **Identification of Na,K-ATPase subunits by Western blotting.** The file shows pictures of uncropped gels used for the immunoprecipitation experiments summarized in Fig. 1 and 3.
(DOCX)
