# Supplementary material for: Oxidative Stress (Glutathionylation) and Na,K-ATPase Activity in Rat Skeletal Muscle
Source: PLoS One. 2014 Oct 13;9(10):e110514. doi: 10.1371/journal.pone.0110514 (PMC4195747; doi:10.1371/journal.pone.0110514)
Supplement: File S1 — Identification of Na,K-ATPase subunits by Western blotting. The file shows pictures of uncropped gels used for the immunoprecipitation experiments summarized in Fig. 1 and 3. (DOCX) [file pone.0110514.s001.docx]

**Supporting information.**

**Identification of Na,K-ATPase subunits by Western blotting.** The main manuscript only contains selected Western blots. The pictures below show uncropped gels used in the immunoprecipitation experiments. Glutathionylated proteins were immunoprecipitated with a GSH antibody, and the Na,K-ATPase proteins identified with Western blots of the immunoprecipitate.





55 kDa

**β 1 isoform**. Samples were applied in pairs (homogenate-immunoprecipate). The Image Quant system also provides a normal-light picture of the gel with visible molecular weight markers (color code, invisible on the gels). This is used to identify the molecular weight on blots like the one shown here. The molecular weight of the dominant band is 55 kDa.

The molecular weight for β1 in homogenates and immunoprecipitates seem to be different. This is probably due to the different amount of protein applied; 10 μg of protein is applied per lane for the homogenates, whereas the amount of protein in the immunoprecipitate is lower (and unknown). It is also a possibility that glycocylation differs.





Direction of protein

Movement

↓

55 kDa

**β2 isoform**. β2 antibodies were used . Only the dominant band at 55 kDa is quantified. Samples were applied in pairs (muscle homogenates and immunoprecipitates).





100kDa

55 kDa

α isoforms.

The gel shown above was also used in the quantification of the glutathionylated α isoforms. The β antibody was applied first and the bands quantified. After that, the α antibody was applied and the new bands identified. The α antibodies labeled some proteins with a lower molecular weight than obtained with the β antibodies. This is considered to be unspecific labelling. Only the band with a molecular weight of 100 kDa was quantified.

The experiments above were repeated in two independent experiments, the second set of gels is not shown.

Figure 3. The gel below is part of the basis for the manuscript Figure 3. One half of each sample was pre-treated with 5 mM GSSG, immunoprecipitated with a GSH antibody and Na,K-ATPase β2 subunits were identified on the gel. Anti β2 antibodies used.


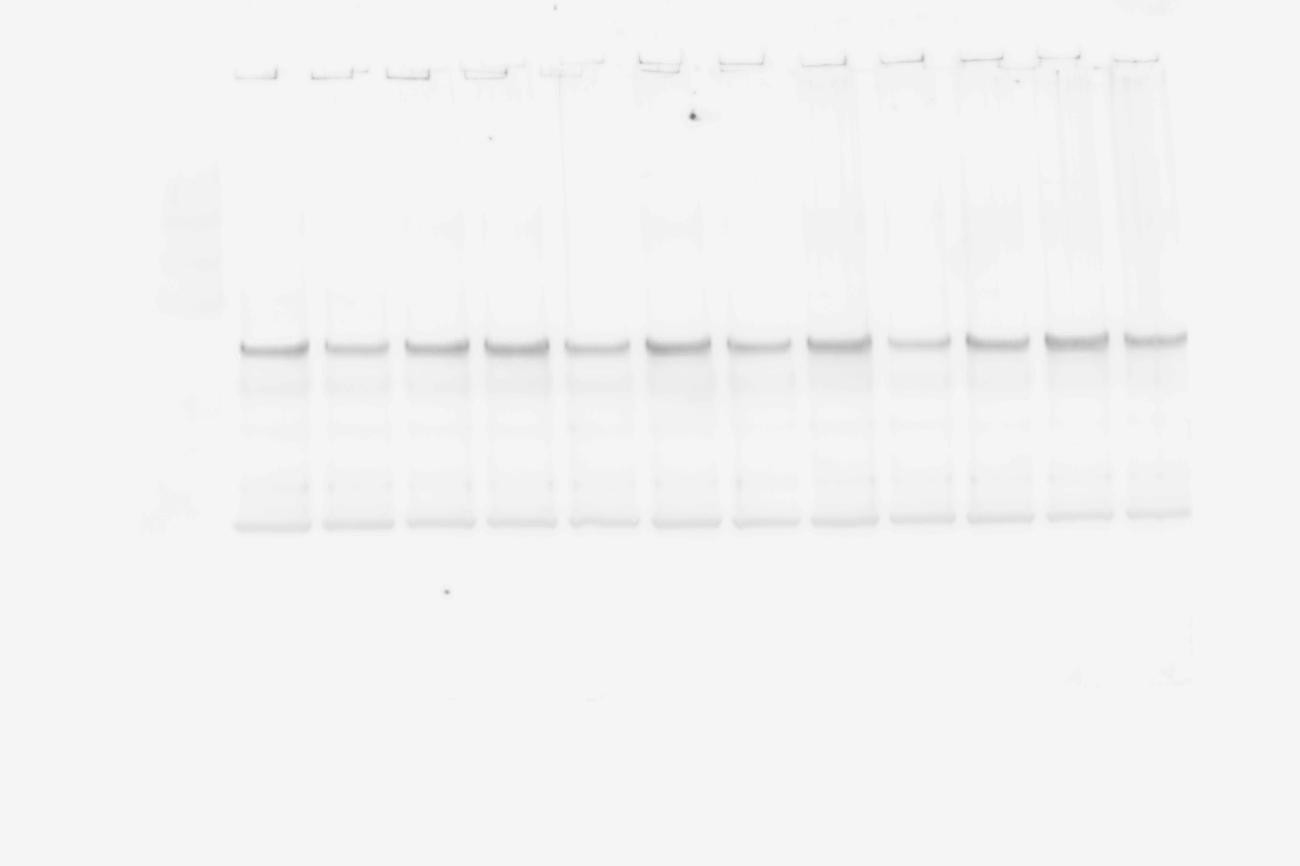


β2 55 kDa
